# Comment on “Optimizing the Delivery of Inhaled Medication for Respiratory Patients: The Role of Valved Holding Chambers”

**DOI:** 10.1155/2019/6475651

**Published:** 2019-07-25

**Authors:** Michael T. Newhouse, Israel Amirav

**Affiliations:** ^1^Firestone Institute for Respiratory Health, St. Joseph's Hospital, McMaster University, Hamilton, ON, Canada; ^2^Pediatric Department, University of Alberta, Edmonton, AB T6G2C6, Canada

McIvor et al. [1] recently suggested that regulatory authorities (e.g., US Food and Drug Administration (FDA), Health Canada, and Japan's Pharmaceuticals and Medical Devices Agency) should harmonize and follow the European Medicines Agency (EMA) recommendation that valved holding chambers (VHCs) are not interchangeable.

We endorse their position but think that their recommendation that devices meet “minimal quality and performance standards” (based only on ex vivo studies that characterise MDI aerosol particle characteristics and dose output) lacks scientific rigor.

We suggest that there are many additional and clinically important factors related to how these devices are used by patients in vivo under real-life conditions that are not, and cannot be, accounted for by laboratory studies such as that of McIvor et al. and previous similar reports [2], since they fail to take into account “real-world” conditions.

We would like to draw attention to three important yet neglected “real-world” aspects that are not considered when comparing VHC and mask aerosol delivery *ex vivo*. These relate to compliance, inspiratory flow, and mask dead space.

With respect to adherence, compliance, and acceptance, rejection of masks has long been known to be a major factor in real-life aerosol therapy of infants and young children [3]. Yet, none of the regulatory bodies takes this critical factor into account. The only mask that specifically addresses this issue is the soft and very low dead-space SootherMask™, specifically designed to accept the child's own pacifier [4], thereby providing a much more familiar, comfortable, and acceptable device for these children. Furthermore, SootherMask™ has been shown to be the only evidence-based method for consistently achieving bronchodilator aerosol therapy to infants while asleep [5]. Previous studies were not able to accomplish this [6], a particularly problematic issue as many children are frequently awakened by asthma exacerbations and require treatment at night.

Another important factor is the inspiratory flow velocity (IFV). Low IFV has been shown to facilitate reduced upper respiratory tract aerosol deposition and considerably increased aerosol delivery to the lungs of about 40%, of even relatively large particles (MMAD 6.5 *µ*m), if the IFV is in the order of about 12 L/min [7]. This will, of course, depend not only on the resistance to flow but also on patient effort. In this regard, patients can benefit from inspiratory flow feedback provided by means of a reed or whistle, yet there are many different such devices and this factor has never been considered in the in vitro studies. For example, most commonly used VHC audio feedback devices use a reed to alert patients at an IFV of 30–60 L/min. By contrast, the newer InspiraChamber® whistle alerts the patient at 15–30 L/min, thus potentially reducing the turbulence-related throat dose, thereby augmenting the lung dose.

Additionally, the extremely important question of mask dead space used in small children is not taken into account in regulatory conclusions. Recent studies have shown considerable differences in dead space with various masks [8]. Clearly, if the volume of the mask dead space is not taken into account and is significantly larger with one brand of mask or if greater pressure is required to achieve a seal [9], thus frightening the child, with even the best in vitro studies showing differences in aerosol delivery, the results may be clinically irrelevant!

In our opinion, using only laboratory criteria to determine superiority of any MDI + VHC combination over another fails to take into account important “real-world” device and patient-related clinical issues. These are determined by pMDI and VHC/mask design features that individually or in combination will surely have an effect that is equal to or greater than the recent laboratory-demonstrated differences in various MDI + VHC combinations [2] that, as a result, have led to totally unwarranted clinical conclusions [1].
